# Role of Sirtuins in Physiology and Diseases of the Central Nervous System

**DOI:** 10.3390/biomedicines10102434

**Published:** 2022-09-29

**Authors:** Justyna Chojdak-Łukasiewicz, Anna Bizoń, Marta Waliszewska-Prosół, Agnieszka Piwowar, Sławomir Budrewicz, Anna Pokryszko-Dragan

**Affiliations:** 1 Department of Neurology, Wroclaw Medical University, Borowska 213, 50-556 Wroclaw, Poland; 2 Department of Toxicology, Faculty of Pharmacy, Wroclaw Medical University, Borowska 211A, 50-556 Wroclaw, Poland

**Keywords:** sirtuins, neurodegeneration, multiple sclerosis, Alzheimer’s disease

## Abstract

Silent information regulators, sirtuins (SIRTs), are a family of enzymes which take part in major posttranslational modifications of proteins and contribute to multiple cellular processes, including metabolic and energetic transformations, as well as regulation of the cell cycle. Recently, SIRTs have gained increased attention as the object of research because of their multidirectional activity and possible role in the complex pathomechanisms underlying human diseases. The aim of this study was to review a current literature evidence of SIRTs’ role in the physiology and pathology of the central nervous system (CNS). SIRTs have been demonstrated to be crucial players in the crosstalk between neuroinflammation, neurodegeneration, and metabolic alterations. The elucidation of SIRTs’ role in the background of various CNS diseases offers a chance to define relevant markers of their progression and promising candidates for novel therapeutic targets. Possible diagnostic and therapeutic implications from SIRTs-related investigations are discussed, as well as their future directions and associated challenges.

## 1. Introduction

Silent information regulators, sirtuins (SIRTs), are a family of enzymes–histone deacetylases which take part in major posttranslational and epigenetic modifications of proteins and thus contribute to multiple processes within cellular environment. They are engaged in metabolic and energetic transformations as well as regulation of the cell cycle, including its maturation and proliferation, viability, and death. SIRTs seem particularly involved in the reaction and compensatory response of the cell to various kinds of stress caused by external factors [1]. Recently, SIRTs have gained increased attention as the object of research because of their wide prevalence in living organisms, multidirectional activity, and possible role in the complex pathomechanisms underlying human diseases. They have aroused special interest in the field of neuroscience [2]. SIRTs have been found to be important players in the development, plasticity, and aging of the nervous tissue. There is growing evidence of their role in substantial functions of the central nervous system (CNS), including cognition, perception of pain, mood and behavior, sleep, and circadian rhythm [3,4,5].

A relevant role of SIRTs has been mainly recognized in neurodegenerative processes with primary neuronal loss. However, these enzymes are also involved in altered metabolism, neuroendocrine regulation, and immune-mediated inflammatory processes, so they may also contribute to other processes resulting in secondary CNS injury [2]. Exploration of SIRTs might provide a better understanding of the nature of various CNS disorders. Moreover, they seem to be promising candidates for markers of CNS damage or repair and also for targets of novel therapeutic interventions [6].

The aim of this study was to review a current literature evidence of the SIRTs’ role in the physiology and pathology of CNS, with possible diagnostic and therapeutic implications for CNS disorders.

## 2. Method—Search Strategy

**Figure 1:**
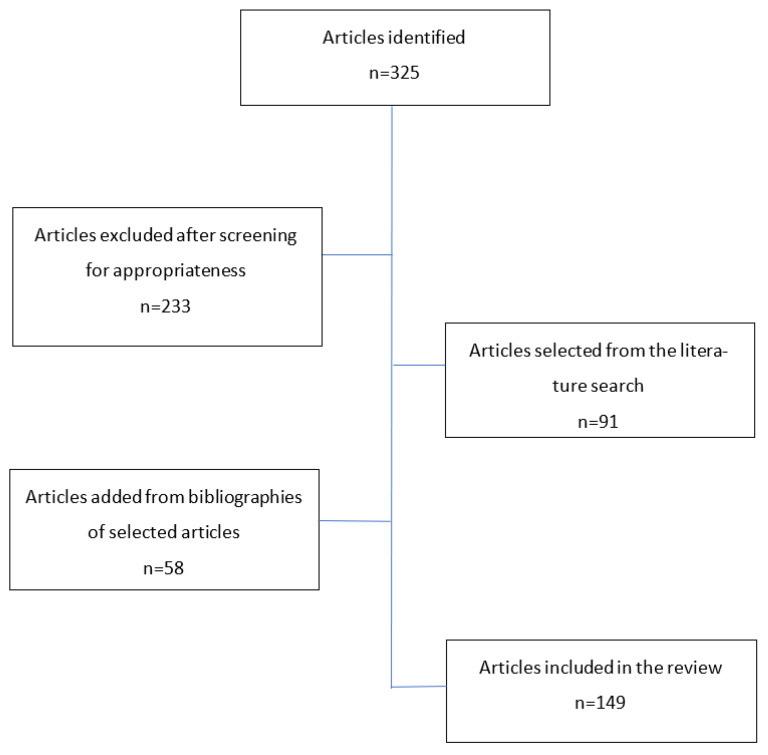
Flow chart of article selection.

A search was conducted using PubMed and Google Scholar, considering relevant data from January 2010 up to June 2022. The following combined key terms were used to search titles and abstracts in the databases: “sirtuins (SIRT)”, “central nervous system”, and “diseases/disorders”. Only reports published in English language with available full text content were included and duplicates were removed. A selection of studies was performed independently in an unblinded standardized manner by two authors. The retrieved papers, as well as their reference lists, were further analyzed for their relevance to the topic. Finally, 149 studies were identified as the most relevant and thus were included in the review. The flow chart of the search strategy is shown in Figure 1.

## 3. SIRTs Functions on a Cellular and Systemic Level

**Figure 2:**
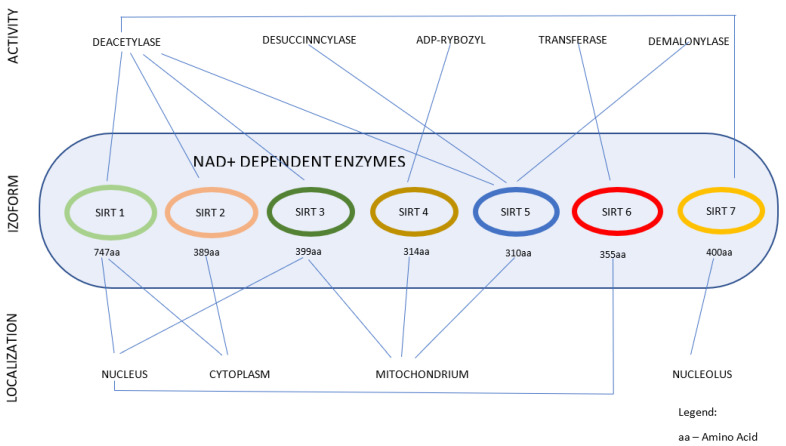
Characterization of sirtuin proteins.

Histone acetylation and deacetylation, catalyzed by histone acetyltransferases (HATs) and deacetylases (HDACs), are crucial processes engaged in modifications of genes expression [7]. HDACs are divided into four classes and SIRTs belong to the III class [8,9,10]. Among these four classes of HDACs, only SIRTs require nicotinamide adenine dinucleotide (NAD+) as a co-substrate to hydrolyze acylated amino acid residues [11]. SIRTs are not only involved in the deacetylation of histones, but also of other proteins, including enzymes or transcription factors [12]. SIRTs are present in all living organisms, ranging from the most primitive Prokaryota (unicellular archaea and bacteria) to the highly organized plants and animals [13]. In the mammalian body, they are widely expressed in the majority of tissues [14]. The multiplicity of genes encoding these enzymes leads to the divergence of isoforms, which differ in their localization in cellular compartments (15). SIRT1, SIRT6, and SIRT7 are located in the nucleus, SIRT2 is mainly found in the cytoplasm, while SIRT3, SIRT4, and SIRT5 are found in mitochondria. In particular conditions, SIRT1 can be transferred to the cytoplasm and SIRT3 can be relocated between mitochondria and the nucleus [1,15]. According to molecular phylogenetic analysis, mammalian SIRTs are divided into four classes (I, II, III, and IV): SIRT1, SIRT2, and SIRT3 belong to Class I, SIRT4 to Class II, SIRT5 to Class III, and SIRT6 and SIRT7 to Class IV [16]. Each SIRT contains 275 amino acid conserved catalytic core domain responsible for the binding of nicotinamide adenine dinucleotide (NAD), as well as the unique sequences at N- and/or C-terminal, which play specific regulatory roles [17]. SIRTs isoforms differ in their enzymatic activity: SIRT1, SIRT2, SIRT3, and SIRT-7 predominantly exhibit properties of NAD-dependent deacetylase; SIRT4 and SIRT6 may act as deacetylases and ADP-ribosyltransferases [2], whereas SIRT5 may act as deacetylase, desuccincylase, and demalonylase [18] (Figure 2). NAD+ plays an essential role as cofactor in the reaction of deacetylation, cleaving a residue acetic acid from protein substrates. ADP-ribosylation takes place during DNA damage/repair by poly (ADP-ribose) polymerases (PARP) proteins [19,20]. Posttranslational ADP-ribosylation, via modification of different acceptor proteins, influences diverse cellular processes, including chromatin organization, transcription, DNA repair, genomic stability, and processes of programmed cell death: apoptosis, necrosis, and autophagy. Generally, SIRTs are activated in the response to various factors which interfere with maintenance of cellular homeostasis, such as oxidative stress (particularly within endoplasmic reticulum), nutritional and metabolic imbalance, or inflammation [21].

SIRTs deacetylating activity is dependent on NAD+, which is an important redox signaling molecule. Therefore, they are involved in the regulation of cellular antioxidant and redox signaling (ARS) pathways [22,23]. SIRT1 and SIRT2 were indicated to play a crucial role in ARS [24]. SIRTs are associated with several of the molecules of the antioxidant response element (ARE), which mediates signaling events involved in transcriptional regulation of gene expression in cells exposed to oxidative stress [22]. For example, SIRT1 inhibits the nuclear factor kappa-light-chain-enhancer of activated B cells (NF-ĸB) signaling pathway by deacetylating its p65 subunit. The reduced concentration of NF-ĸB decreases the production of reactivity oxygen species (ROS). Furthermore, the inhibition of NF-ĸB results in decreased activity of cytochrome p450, NADPH oxidase, and xanthine oxidoreductase. At the same time, SIRTs can induce activity of the most important antioxidant enzymes: superoxide dismutase (SOD), which converts the anion superoxide in hydrogen peroxide [25], or S-transferase glutathione, one of the most important detoxifying enzymes [26]. Moreover, it was confirmed that modulation of NF-ĸBs affects activity of inflammatory mediators and acts as a regulator of different metabolic pathways, such as gluconeogenesis, glycolysis, and lipid metabolism. SIRT1 affects cell metabolism, especially of glucose and lipids, in multiple ways, e.g., regulating glucose concentration, insulin secretion, adipose biogenesis, and myogenesis. Its effect on gluconeogenesis is ambiguous. When SIRT1 deacetylates CREB-regulated transcription co-activator 2 (CRTC2), which affects CRTC2 degradation, the production of hepatic glucose is deceased. Whereas, when SIRT1 activates forkhead transcription factors (FoxO1) and peroxisome proliferator-activated receptor-γ coactivator (PGC-1 α), hepatic glucose production is increased. SIRT1 could also induce FoxO1-dependent transcription of genes important for hepatic glucose production, which increases glucose concentration. Moreover, glycolysis was shown to be suppressed by SIRT1 via different pathways, and transgenic mice with overexpression of SIRT1 presented with greater insulin sensitivity than the control ones [27].

SIRTs also play a crucial role in cellular homeostasis and apoptosis, mainly due to the influence on p53 and FoxO functions. SIRT1 regulates two types of p53-mediated apoptotic mechanisms: dependent or independent on p53 transcription [28]. SIRT1 deacetylates FoxO3 and/or FoxO4, thus attenuating FOXO-induced apoptosis and potentiating FOXO-induced cell-cycle arrest. Furthermore, SIRTs located in mitochondria are implicated in mitochondrial pathway of apoptosis. It was shown that SIRT3 plays a pro-apoptotic role in both BCL2-53- and c-Jun N-terminal kinase 2 (JNK)-regulated apoptosis [29]. SIRT6 is mainly involved in DNA repair due to its ADP-ribosyl transferase activity. PARP1 is a poly-ADP ribose polymerase associated with response to DNA damage, which plays a relevant role in DNA replication, single- and double-stranded break repair, and degradation.

On a systemic level of the human organism, SIRTs appear as relevant players in its development, ageing, and longevity, in regulation of innate and adaptive response of the immune system, as well as in metabolic processes which provide maintaining homeostasis in changing conditions of environment. Thus, the role of SIRTs has been recently extensively sought in a range of pathological conditions, including metabolic and endocrine disorders, neoplasms, and diseases of the cardiovascular, gastrointestinal, and nervous system [1,4].

## 4. SIRTs in the Central Nervous System

The presence and activity of SIRTs in the CNS was demonstrated mainly for SIRTs 1–5 (particularly SIRT1 and SIRT2 seem to predominate in mammalian brain), while SIRT6 and 7 play a more relevant role in other tissues [1]. SIRTs can be found in different types of cells in the nervous tissue: SIRT1 is expressed in neural stem and progenitor cells, as well as mature neurons, but also in microglia and astrocytes [30,31], SIRT2 occurs mainly in oligodendrocytes and paranodal areas of myelin sheath [32,33], and SIRT4 occurs mainly in the late embryonic stage of neurons [34]. Distribution of SIRTs throughout CNS structures may also vary: SIRT1 has been mainly detected in the hippocampus, neocortex, cerebellum, and hypothalamus, SIRT2 in the spinal cord, brain stem, hippocampus, and striatum [32], and SIRT5-in the brain stem and cerebellum.

SIRTs appear to be key players in the processes of maturation, viability, and death of the nervous tissue. In experimental studies, SIRT1 and SIRT2 were reported to be involved in signaling pathways which mediate differentiation of mesenchymal stem cells or progenitor cells into neurons and oligodendrocytes [35]. Investigations on animal models of demyelination showed that SIRT2 plays an important role in formation of myelin sheath and its interaction with axons [36]. Findings from knockout studies indicated that SIRT1 or SIRT3 activation or SIRT2 inhibition provide protection for healthy neurons exposed to adverse factors due to stimulating autophagy, as well as inhibiting oxidative mechanisms and apoptotic pathways induced by FoxO, NF-ĸB, and p53 signaling. The anti-oxidative effect is further supported by SIRT4 and 5, which modulate mitochondrial homeostasis [6]. SIRT1 and 3 were also shown to suppress inflammatory response in the neuronal environment (particularly expressed by microglia) of murine cell lines [37]. With ageing, decreased levels of SIRT1, 3, and 4, and accumulating isoforms of SIRT2 are supposed to promote inflammation, energetic deficit, and apoptotic processes, leading to neurodegeneration and cell death [6]. These findings suggest the important role of SIRTs in the physiological ageing and pathological degeneration of the brain.

Further studies on animal models provided evidence for the SIRTs role in advanced functions of CNS neuronal networks, responsible for cognition, mood, and behavior. It has been demonstrated that SIRT1 is indispensable for regular synaptic function and plasticity, associated with complexity and branching of neuronal dendritic arbors, which is essential for processes of memory and learning [37]. Interaction of SIRT3 with the long chain acyl-CoA dehydrogenase (LCAD) seems to be involved in regulatory mechanisms modulating animals’ behavior conditioned by chronic stress and is thus associated with stress resilience and depressive-like reactions [38]. Multiple pathways engaging SIRT1 and SIRT3 activity (i.e., acetylation of FoxO1, modulation of SOD, interaction with insulin-like growth factor (IGF-1) and 5’AMP-activated protein kinase (AMPK) contribute to the neuroendocrine function of hypothalamus/pituitary axis, including regulation of nutrient sensing, weight gain/loss, and behavior associated with food intake [6,39,40]. Furthermore, relevant links were demonstrated between NAD-dependent deacetylase function of SIRT1 and expression of the genes associated with circadian rhythm control in young and aged animals, indicating the potential role of SIRTs in the background for the sleep–wake cycle and its decay with aging [4].

## 5. SIRTs in CNS Diseases

### 5.1. Traumatic Brain Injury

Traumatic brain injury (TBI), due to the type and intensity of head contusion, may result in symptoms ranging from mild and transient concussion to severe permanent neurological deficit. Primary injury, immediately following trauma, leads to mechanical disruption, structural deformation, and necrosis of cerebral tissues. Secondary injury is associated with emerging cascade of events within neurons and glial cells, including oxidative stress, excitotoxicity, and mitochondrial dysfunction, which results in diffuse neuronal and axonal damage [41]. Furthermore, the injury of neurons and astrocytes triggers inflammatory processes, enhanced by disruption of the blood–brain barrier and the release of proteins such as glial fibrillary acidic protein (GFAP), myelin basic protein (MBP), neuron-specific enolase (NSE), and tau protein [41]. SIRTs, as modulators of processes associated with cell death and repair, have been investigated for their potential role in diminishing the results of TBI. It was demonstrated on animal models of TBI that expression of SIRT1 gradually increases following brain injury. Moreover, inhibition of SIRT1 promoted mitochondrial damage and induced apoptosis via p38 MAPK signaling, particularly extracellular signal-regulated protein kinases 1 and 2 (ERK1/2). Thus, SIRT1 might provide neuroprotective response to brain injury due to a reduction of mitochondrial stress and regulation of autophagy [40]. There are conflicting results about SIRT2’s role in TBI-related processes. SIRT2 inhibits p-53-mediated ferroptosis (iron-dependent programmed cell death pathway, associated with accumulation of lipid peroxides), which constitutes one of the TBI components [42]. Moreover, inhibition of SIRT2 led to BBB disruption and exacerbation of posttraumatic brain edema [40,42]. On the other hand, SIRT2 inhibition was shown to reduce the levels of proinflammatory cytokines (IL-1β, IL-6, TNF-α, MIP-2, and MCP-1), as well as blocked nuclear translocation of NF-κB and pathways involved in activation of autophagy [3,40]. Modulation of SIRT1 and 2 activity, through reduction of oxidative stress and inflammatory cascade, might provide a potential target for therapeutic interventions in TBI.

### 5.2. Cerebrovascular Disease

Ischemic stroke is the result of a sudden decrease in blood supply to the brain, due to thrombosis, embolism, or systemic hypoperfusion. The current treatment of ischemic stroke is based mainly on interventions which cause reperfusion, while there is still no evidence of effective neuroprotective agents, capable of saving the brain tissue from permanent ischemic injury. Prolonged and uncompensated brain ischemia, with deprivation of oxygen and energy failure, initiates the release of excitatory neurotransmitters, followed by the formation of reactive oxygen species and gene expression changes, accompanied by induction of inflammation, which ultimately causes necrotic cell death and irreversible tissue damage. Loss of vascular integrity leads to the breakdown of the BBB, which contributes to inflammatory components of ischemic brain injury and to the development of cerebral oedema [43]. Almost all SIRTs, especially SIRT1 and 3, have been found to be involved in the processes following cerebral ischemia [44].

SIRT1 seems to play an essential neuroprotective role in acute stroke. Raval et al. [45] and Hattori et al. [46] demonstrated that animals with a lower level of SIRT1 presented with the worst outcomes after MCA occlusion and had a larger infarct volume, associated with significantly increased acetylation levels of NF-κB, while the overexpression of SIRT1 was connected with milder degree of post-stroke damage [46,47]. Similar results were observed by Wang et al. [48] and Hernandez-Jiménez et al. [49]. In clinical study of Esmayel et al. [50], the plasma levels of SIRT-1 were significantly lower in the patients with acute cerebrovascular events than in the healthy control group. The neuroprotective role of SIRT1 was suggested to be linked with downregulation of NF-κB pathway and mitochondrial biogenesis via PGC-1α activation. Additionally, SIRT1 activation is connected with deacetylation and upregulation of hypoxia-inducible factor-2alpha (HIF-2α), which stimulates production of erythropoietin and initiates the cell survival process [51]. Another probable mechanism is associated with adenosine monophosphate (AMP)-activated kinase (AMPK) pathway, a main regulator of cell energetic processes. The SIRT1-dependent AMPK pathway activation was related with the survival of neurons during ischemia [52].

SIRT2 exhibits a negative impact on ischemic brain injury, as one of the mediators in oxidative stress-induced cell death. SIRT2 also activates FOXO3a, which promotes the expression of pro-apoptotic proteins [53]. SIRT3, similarly to SIRT1, contributes to neuroprotection by upregulating proteins responsible for reactive oxygen species (ROS) detoxification (e.g., catalase and superoxide dismutase (SOD) and mitochondrial biogenesis (e.g., NRF1 and ERRs). Wang et al. [54] suggested that SIRT3 protects neurons from hypoxic injury via pathways which involve PGC-1α (peroxisome proliferator activated receptor (PPAR)-γ co-activator 1-α) and manganese SOD. Interactions between SIRT3 and PGC-1α depend on the level of oxygen and glucose supply to the tissues, so they provide mechanisms that compensate for cerebral ischemia [54]. Neuroprotective properties of SIRT3 are also associated with the mediation of ketone body metabolism [55]. In addition, SIRT1-SIRT3 axis was found to regulate BBB permeability during cerebral ischemia, preventing vascular oedema [56]. The protective role of SIRT4 in ischemic stroke is supposed to be associated with its anti-excitotoxic effects, mainly by promotion and maintaining adequate level of glutamate transportation via upregulation of glutamate transporter-1 (GLT-1) [40,57]. SIRT5 cooperates with SIRT3 in supporting mitochondrial function through deacetylation or desuccinylation of appropriate proteins. The anti-oxidative and anti-apoptotic activity of SIRT5 in ischemic stroke is probably linked with PKCε activation, but the exact signaling pathways involved have not been yet recognized [58]. The reduced expression of SIRT6, reported during ischemic stroke [59,60], was associated with stabilization of NF-κB pathway and increased secretion of pro-inflammatory cytokines, such as HMGB1 (high mobility group box). SIRT6 also suppresses phosphorylated-protein kinase B (p-Akt, at Ser473), which leads to necrotic cell death due to disinhibition of the autophagy and possibly via FOXO3a pathway [61]. SIRT7 has been identified as a negative regulator of HIF-1α and HIF-2α, which are transcriptional factors that are essential in mediating adaptive responses to hypoxia [62].

In cases of intracerebral or subarachnoidal hemorrhage (ICH, SAH), caused by bleeding within the brain parenchyma or meninges, the primary injury is caused by mechanical damage to the brain tissue resulting from the volume of extravasated blood. The following secondary brain damage and necrosis develops due to cytotoxicity of blood compounds and includes elements of excitotoxicity, oxidative stress, and inflammation. It was shown that activation of SIRT1 in mice with ICH was associated with milder neurological deficit and reduced expression of proinflammatory cytokine, IL-1β [63]. Increase in SIRT1 activity in brain cortex, with the peak at 24 h after experimental SAH in rats, indicated its potentially protective role against early brain injury [64]. Diminished expression of SIRT5 expression, as well as decreased activity of succinylated citrate synthase, was demonstrated during SAH in rats, which suggested that SIRT5 protects mitochondrial metabolism and supports maintaining energetic homeostasis [65].

Understanding the role of SIRTs in pathomechanisms of ischemic or hemorrhagic brain injury stimulated attempts of therapeutic interventions targeted at these enzymes. Resveratrol, a natural polyphenolic compound that activates SIRT1, was found to provide neuroprotective effects in animal models of focal and global cerebral ischemia [45,66], presumably due to downregulation of mitochondrial uncoupling protein 2 (UCP2) [67]. Another SIRT1 activator, A3, decreased the volume of experimental infarct (on the contrary to SIRT1 inhibitor sirtinol), as well as reducing early brain injury in SAH [3]. The use of recombinant SIRT3 resulted in reduction of brain necrosis due to ischemia, correlated with decreased ROS production and elevated levels of PGC-1α and manganese SOD. These findings encourage further studies to explore the possibility of using SIRT modulators as neuroprotective treatment options for strokes. It is also worth highlighting that a relevant role of decreased concentration of NAD due to activity of SIRTs was established in cardiovascular diseases (hypertension, cardiac arrhythmias, cardiomyopathy) and metabolic syndromes (including dyslipidemia and diabetes), which are major risk factors for cerebral stroke [68]. Thus, modulation of selected SIRTs activity, explored as potential therapeutic strategies for these disorders, might also contribute to more effective prevention of strokes [69].

### 5.3. Neurodegenerative Disorders

Neurodegenerative diseases are associated with progressive neuronal loss within particular areas of CNS, which determines their clinical presentation and chronic progression of neurological deficit. According to the current views upon these disorders, intra- and extracellular accumulation of misfolded proteins constitutes the major element of their background. These aggregates initiate cascade of events, including free radical formation and oxidative stress, mitochondrial dysfunction with emerging energetic deficit, and secondary neuroinflammation mediated by microglia. The resulting dysfunction of neuronal transport and signaling, as well as the activation of apoptotic pathways, ultimately lead to irreversible damage to CNS [70].

#### 5.3.1. ALS (Amyotrophic Lateral Sclerosis)

ALS is a neurodegenerative disease characterized by selective degeneration of the upper and lower motor neurons in the brain and spinal cord. ALS is associated with rapid progression of disability (including limbs paresis, dysarthria, and dysphagia) and an invariably fatal outcome within a few years due to respiratory failure. Few available treatment options only mildly prolong the progression of the disease. In familial forms of ALS, a number of mutations in various genes have been identified, including SOD1, TAR DNA-binding protein 43 (TDP43), fused in sarcoma (FUS), ubiquilin proteins (UBQLN2), and chromosome 9 open reading frame 72 (C9ORF72) [71,72]. However, most ALS cases are sporadic, with the background predominantly including mitochondrial dysfunction and oxidative stress.

There is growing evidence for a link between activity of SIRT1 and SIRT3 and background of ALS [71]. Altered expression of these SIRTs correlates with the course of disease in animal models of ALS, e.g., mutant mice with higher neuronal expression of SIRT-1 achieved longer lifespan [73]. In other experimental studies [74], a decrease of SIRT3 expression in brainstem and spinal cord, with an increase in mitochondrial form of the enzyme in muscles, was observed with progression of the disease [3]. Both sirtuins are known as regulators of mitochondrial function, and the hypothesized mechanisms of their protective action in ALS include regulation of mitochondrial ROS production [75]. SIRT1 has also been shown to deacetylate HSF 1, which increases transcription of chaperones (like HSP70 and HSP 25), supporting intracellular protein homeostasis and reducing toxicity [76,77]. Another activity of SIRT1 and SIRT3, deacetylation of PGC1α, can affect biogenesis of mitochondria and provide their integrity [78,79]. Activating SIRT1 (e.g., using resveratrol or initiating lentivirus infection) allowed neurodegeneration to diminish in mouse model of ALS [3,80,81]. Further studies are necessary to investigate SIRT1 as a potential target for therapeutic interventions in patients with ALS.

#### 5.3.2. Parkinson’s Disease

Parkinson’s disease (PD) is one of the most common neurodegenerative diseases, predominantly characterized by motor deficit; its cardinal features include bradykinesia, rigidity, rest tremor, and loss of postural reflexes. Non-motor symptoms of PD, co-occurring throughout the disease, comprise olfactory dysfunction, dysautonomia (e.g., orthostatic hypotension, urogenital dysfunction), neuropsychiatric issues (cognitive impairment, depression, behavioural disturbances), and sleep disorders. PD background is associated with progressive loss of neurons in *substantia nigra* and subsequent failure of the dopaminergic transmission within basal ganglia. There are a range of therapeutic options aimed at restoration of dopaminergic neural circuits. However, due to the progressive and long-lasting course of the disease, as well as wearing-off and other adverse effects of medications, effective treatment of PD still remains a challenge [82]. In a small proportion of PD cases, genetic background has been identified, with β-Glucocerebrosidase (GBA), leucine-rich repeat kinase 2 (LRRK2), parkin (PRKN), protein/nucleic acid deglycase (DJ1) and phosphatase and tensin homologue (PTEN)-induced putative kinase 1 (PINK1) as the most common mutations [70]. Pathologically misfolded α-synuclein seems to be the main factor initiating neurodegenerative process in PD, and it is abundant (together with heat shock proteins and ubiquitin) in Lewy bodies (LBs)–neuronal inclusions, constituting the pathological hallmark of the disease [83].

SIRTs seem to play complex and bidirectional roles in the processes underlying PD background [84]. Reduced activity of SIRT1 was reported in autopsy brain tissue specimens from PD patients [85], as well as in pluripotent stem cell-derived dopaminergic neurons in PD cases with LRRK2 mutation [86]. Administration of SIRT1 activator resveratrol or overexpression of PGC-1α, deacetylated by SIRT1, was found to decrease degeneration of dopaminergic neurons in the mouse model of PD [87]. These findings implicated that SIRT1 neuroprotective properties are associated with regulation of the autophagy, mitochondrial function, and neuroinflammation [74,75,76]. However, some studies suggested that SIRT1 fails to prevent damage to dopaminergic neurons, especially mediated by tyrosine hydroxylase, which may indicate variable effects of SIRT1 expression depending on the conditions [88]. SIRT2 mediates various processes involved in PD pathogenesis, including α-synuclein aggregation, microtubule function, oxidative stress, inflammation, and autophagy, but with opposed effects upon the disease outcomes [89]. On one hand, SIRT2 regulates autophagy processes and accelerates clearance of misfolded α-syn aggregates, reducing their toxicity. The absence of SIRT2, conditioned by rotenone-induced oxidative stress, was shown to promote α-syn aggregation [85]. On the other hand, diminished expression of SIRT2 deacetylating α-synuclein suppresses aggregation of the altered form of this protein [90].

Activity of SIRT2 is also connected with enhanced deacetylation of tubulin, which causes impairment of microtubules and disturbances in axonal kinetics and transport [91]. Furthermore, SIRT2 was found to aggravate the damage of nigrostriatal dopaminergic neurons by enhancing apoptotic pathways (deacetylation of FOXO3a and induced expression of Bim factor) [91,92]. These results suggest that pharmacological inhibition of SIRT2 may have a beneficial, neuroprotective effect in PD. The mitochondrial sirtuins (SIRT3-5) exhibit neuroprotective effect in PD mainly by regulation of the mitochondrial respiratory chain and preservation of mitochondrial antioxidant capacity, as well as promotion of autophagy [93,94]. Expression of SIRT6 is increased in PD and associated with production and secretion of proinflammatory cytokines (e.g., TNF-a) [95]. Thus, SIRT6 seems involved in inflammatory component of neurodegeneration, which enhances damage to dopaminergic pathways. The evidence from experimental studies suggests that activators of SIRT1 (resveratrol or oxyresveratrol) and substrate competitive SIRT2 inhibitors are promising candidates for neuroprotective agents beneficial in PD [94,96].

#### 5.3.3. Alzheimer’s Disease (AD)

Alzheimer’s disease (AD) is the most frequent cause of dementia-progressive deterioration of multiple cognitive domains, which severely affects patients’ social functioning and many aspects of daily living. The pathology of AD is associated with extracellular accumulation of amyloid β plaques and intracellular formation of neurofibrillary tangles, composed of hyperphosphorylated forms of the microtubule-associated protein tau [97]. In rare inherited cases of AD (1–2%), mutations were found in the genes encoding amyloid-beta precursor protein (APP) and presenilins; other genetic factors (alleles of apolipoprotein ε4 or TREM genes) may also increase the risk of disease. In the vast majority of sporadic cases, the reason for misfolded protein aggregations has not been elucidated. Abnormal protein oligomers initiate a cascade of events, resulting in diffuse neuronal damage. The loss of neurons, predominantly cholinergic ones, is noted within the limbic system (e.g., hippocampus) and the cerebral cortex. Therapeutic options include modulation of neurotransmission, but effective treatment preventing devastating disease progression is still lacking [82].

SIRTs have been extensively studied in the context of AD background. The results of a study by Pradhan et al. first indicated that serum levels of SIRT1, SIRT3, and SIRT6 were significantly decreased in AD subjects in comparison with those with mild cognitive impairment and healthy controls [98]. Increased expression of SIRT1 was shown to reduce amyloid plaque formation in animal models of AD [3,99,100]. SIRT1 suppresses aβ aggregation and neurotoxicity, i.e., by deacetylation and coactivation of the retinoic acid receptor β, which activates ADAM10 [18], and inhibition of microglial expression of NF- κB [30]. Other putative mechanisms of SIRT1 activity include promotion of non-amyloidogenic processing of amyloid precursor protein via induction of Notch receptor cleavage and/or by decreasing levels of serine/threonine Rho kinase ROCK1 [101]. SIRT1 appears to also be involved in reduction of neurofibrillary tau pathology [102,103] and regulation of neuronal energy metabolism. Furthermore, activation of the SIRT1/AMPK pathway was demonstrated to be linked with biosynthesis of cholesterol and pathogenic impact of APOE 4 upon APP and tau aggregation [104,105]. Activation of SIRT1 was also shown to enhance the integrity of BBB and induce adaptive immune responses which might regulate inflammatory component of degeneration and promote brain resilience [106]. The role of SIRT2 in AD pathogenesis was also investigated. Inhibition of SIRT2 interfered with aggregation of amyloid β and tau phosphorylation, restoring stabilization of microtubules through regulation of autophagy processes, and allowed the clearance of toxic protein oligomers [107,108]. Moreover, inhibition of SIRT2, together with activation of SIRT1, was found to positively regulate synaptic plasticity (especially through modulating long-term potentiation in the hippocampal region). These findings seem particularly interesting in view of hypotheses which emphasize abnormal neuronal excitability and maladaptive synaptic plasticity as core elements of Alzheimer’s disease background [109,110].

SIRT3 expression was found to be decreased in the cerebral cortex of AD patients, which was associated with p-53-mediated mitochondrial and neuronal damage [111]. The overexpression of SIRT3, e.g., upregulated by calorie restriction, prevented neuronal damage caused by ROS formation and oxidative stress [112]. Diminished expression of SIRT6, also detected in AD, was similarly correlated with toxic tau phosphorylation and amyloid β aggregates. Apart from p-53-mediated pathway, SIRT6 was suggested to prevent consequences of naturally accumulating DNA damage [113,114].

The results of interventions activating SIRT1 and 3 or inhibiting SIRT2 were primarily investigated on animal models of AD. Furthermore, formulations of resveratrol (SIRT1 activator) have been tested in a few clinical trials with AD patients. A decrease in plasma and CSF amyloid β concentration was observed following treatment with resveratrol vs. placebo [115]. In another trial, resveratrol was shown to diminish inflammatory markers in CSF [106]. However, the study on peripheral SIRT2 mRNA neither showed differences between AD patients and healthy elderly subjects nor confirmed a correlation between SIRT2 mRNA expression and cognitive performance [116]. These discrepancies illustrate difficulties with translation of experimental findings into clinical trials and indicate the need for further attempts in this field.

#### 5.3.4. Huntington’s Disease (HD)

Huntington’s disease is manifested by involuntary movements (chorea), psychiatric disturbances, and cognitive impairment. The condition is inherited in an autosomal dominant pattern and results from mutations of a huntingtin gene, located on the short arm of chromosome 4. It is caused by expansion of an unstable CAG repeat (its number determines occurrence and severity of clinical presentation), which codes for a stretch of glutamine residues, affecting the conformation and aggregation propensity of huntingtin (mHTT) [117,118]. The mHTT initiate neurodegenerative cascade with transcriptional dysregulation, oxidative damage, and excitotoxicity. Therapeutic options for HD include symptomatic treatment (control of movement disorders, anti-psychotic, and mood-stabilizing agents), with no effect on disease progression. An unfavorable outcome of HD is further enhanced with a high risk of suicide.

The role of SIRTs has been initially investigated on animal models of HD. Neuroprotective properties of SIRT1, reducing mHTT neurotoxicity, were demonstrated in some studies [95,119]. One possible mechanism is attributed to deacetylating TORC1, facilitating its interaction with cAMP-response element binding protein (CREB), which is linked to brain-derived neurotrophic factor (BDNF) and dopamine- and cAMP-regulated phosphoprotein, Mr 32 kDa (DARPP32) expression in neurons. Moreover, inhibition of SIRT1 leads to the decreased deacetylation and altered activity of Foxo3a, p53, and PGC-1α10 proapoptotic pathways [18]. The role of SIRT2 in HD is mostly associated with its involvement in metabolism of cholesterol. SIRT2 may increase the deacetylation of SREBP-2 and its translocation to the nucleus, increasing the biosynthesis of cholesterol, which affects synapse maintenance and contributes to their disruption. A specific SIRT2 inhibitor, AK-1/7, allows the normalization of cholesterol levels in the neurons expressing mHTT [120]. SIRT3 level is increased in patients with HD, presumably due to responses to high level of oxidative damage. SIRT3 regulates the majority of the mitochondrial acetylome and controls mitochondrial dysfunction induced by mHTT; its activation with viniferin promotes resistance to oxidative stress and liver kinase B1 (LKB1) [121]. The role of SIRT4 and 6 in HD is probably associated with the reduction of pyruvate dehydrogenase complex (PDH) complex activity [119].

The investigation of SIRTs’ therapeutic potential in HD focused on modulation of SIRT1 and SIRT2 activity. On animal models of HD, inhibition of SIRT2 reduced the amount of mHTT neuronal inclusions and affected lifespan in mice [122]. Administration of resveratrol and quercetin (activators of SIRT1) was shown to prevent polyglutamine-induced degeneration in striatal neurons [123]. Based on these findings, clinical trials have been designed, testing the efficacy and safety of selisistat, a selective SIRT1 inhibitor in early-stage HD subjects. Some improvements were noted in cognitive and neuropsychiatric measures after the administration of selisistat, and the agent was found to be safe and well-tolerated [124].

#### 5.3.5. Multiple Sclerosis

Multiple sclerosis (MS) is a chronic CNS disease with a complex, predominantly autoimmune-mediated background. The main processes involved in MS etiology include exacerbating inflammatory demyelination and slowly developing degeneration with axonal loss. Multifocal lesions within the brain and spinal cord cause a variety of symptoms of neurological deficit, which result in disability accumulating throughout the disease [125]. Although in recent years great progress has been made in the field of MS diagnosis and treatment, there are still some unmet needs. Several available disease-modifying therapies are targeted mainly at inflammatory component of MS pathology and allow the reduction of immune-mediated activity of the disease. However, significant individual differences in MS course and response to treatment, with a lack of sensitive biomarkers, impede choice of therapy and effective control of MS-related CNS injury. Furthermore, there are only a few options which address the neurodegenerative component of MS background and prevent progressive phases of the disease.

SIRTs deserve attention regarding MS pathology as relevant regulators of the main processes involved in the background of the disease: (auto)immune response, neurodegeneration, and metabolic pathway [126]. The activity of SIRTs was extensively investigated in experimental autoimmune encephalomyelitis (EAE), which is a widely used animal model of MS, and more recently also in clinical studies involving MS subjects. Overexpression of SIRT1 allowed for the reduced death of retinal ganglion cells and axons in the spinal cord in EAE mice, preventing impairment of their visual and motor function [127,128]. Downregulation of SIRT1, controlled by microRNA 32 (miR-32), was shown to increase the production of proinflammatory cytokines such as TNF-α and lymphotoxin in B cells [129]. SIRT-1 plays a regulatory role in microglia activation and suppresses neuroinflammation (mediated by growth factors, chemokines, and cytokines), mainly via inactivation of NF-κB p65 pathway and/or FOXO3 activation [130,131]. There is also some evidence of interaction between SIRT1 and activator protein 1–transcription factor in macrophages; increased expression of SIRT1 is associated with diminished mRNA levels of Cyclooxygenase-2 and production of prostaglandin E [64]. However, some contradictory effects were demonstrated for SIRT1 in EAE models. Its expression was elevated in GFAP-positive cells around EAE lesions, causing the suppression of neuronal progenitor cells proliferation and differentiation into astrocytes [31]. SIRT inhibition or inactivation was demonstrated to ameliorate activity of pro-inflammatory T helper 17 (Th17) cells as well as neurological deficit (onset of paralysis) and was attributed to induced remyelination [132]. Tegla et al. reported increased expression of SIRT1 (with concomitant presence of immunocompetent cells) in acute and chronic demyelinative plaques in comparison with normal brain tissue in post-mortem MS specimens [133]. In the clinical study, MS patients presented with higher plasma levels of SIRT1 than healthy controls [134]. Furthermore, expression of SIRT1 on peripheral blood mononuclear cells not only differentiated MS subjects from healthy controls but also those with MS relapse (exacerbation) from the ones in remission (stable phase of the disease) [135,136]. Thus, SIRT1 seems a promising candidate for markers of the disease activity. SIRT2 is known to plays a relevant role in differentiation of oligodendrocytes, formation of myelin sheath, and its interaction with axons. Deacetylating activity of SIRT2 is attributed to modulating expression of myelin-specific genes as well as function of tubulin, which promotes the arborization of axons [137]. On the other hand, mutations which inhibit SIRT2 activity were shown to block the expression of inflammatory cytokines, and agents suppressing SIRT2 cause a decrease in LPS-induced expression of neuroinflammation genes in microglia in vivo by blocking the nuclear translocation of NF-κB [138]. The diminished level of SIRT2 isoforms was reported in brain tissue specimens from EAE animals as well as in autopsy findings from MS subjects (within demyelinative lesions but not in non-affected cerebral white matter) [139]. Interestingly, antibodies against SIRT2 were found in CSF of MS patients significantly more often than in healthy controls [140]. The mitochondrial sirtuins (SIRT3, SIRT4, and SIRT5) are responsible for protection against oxidative stress and excitotoxicity, which contribute to the neurodegenerative component of MS pathology. Mitochondrial dysfunction, as well as abnormal glutamate metabolism, were postulated as relevant links with SIRTs activity, with the absence of glutamate dehydrogenase (GDH) expression and reduced levels of SIRT3 observed in MS lesions in autopsy findings [141]. In addition, SIRT3 takes part in the regulation of inflammatory processes, promoting secretion of IFN-γ and IL-8 [142]. SIRT4 likely mediates the reaction of ADP-ribosylation and of glutamate dehydrogenase, which reduced glutamate catabolism, leading to its increased level. Inkster et al. showed relationships between mitochondrial-related gene variants in SIRT4 and SIRT5 and neuroimaging correlates of degeneration and axonal loss in MS [143]. SIRT6 plays a multifaceted role in the regulation of immunity processes. It regulates tumor necrosis factor α (TNF-α) release (at transcriptional and post-translational level) and affects secretion of other pro-inflammatory cytokines, such as IFNγ and IL8. Inhibition of SIRT6 was found to reduce the production of IFNγ and IL12 and increased the level of the anti-inflammatory cytokines such as IL-10 [144]. Furthermore, SIRT6 promotes differentiation and maturation of myeloid (but not plasmacytoid) conventional dendritic cells (DCs). In an experimental study, the transcription level of SIRT6 was significantly increased during the chronic stage of EAE [31]. Inhibition of SIRT6 delayed EAE onset by early downregulation of CD40 expression on DCs and T cells infiltration in the CNS. In studies on MS subjects in the earliest phase of disease, presenting with high levels of DCs in peripheral blood, SIRT6 inhibition was demonstrated to interfere with DCs migration and delay conversion from clinically isolated syndrome (first clinical manifestation) to the relapsing-remitting stage of disease [126]. The role of SIRT7 in MS has been less recognized. Burg et al. suggested that SIRT7 could regulate cell differentiation, cytokine production (especially IFNγ) and accumulation of regulatory T cells, but these effects did not modify the clinical course of EAE in mice [145].

The therapeutic potential of SIRTs targeted intervention was investigated on EAE models. Resveratrol as an activator of SIRT1 attenuated neuronal damage and axonal loss [146]. A neuroprotective effect (regarding immune-mediated damage of CNS and behavioral measures) was reported for modulators of AMPK/SIRT1 signaling pathway (linagliptin, lovastatin, and adiponectin) [85,147]. Agents which enhanced SIRT2 expression were shown to enhance protection of axonal integrity and induction of oligodendrocyte maturation, as well as ameliorate clinical signs of neurological deficit [14,148]. Unique findings from the clinical study showed that MS subjects, who responded well to treatment with glatiramer acetate, presented with higher SIRT1 expression than non-responders. Although SIRT-targeted molecules have not been directly investigated in MS clinical trials so far, they might be considered as agents complementary with currently used disease modifying drugs in the future, or as markers of the response to treatment with these [127,146].

## 6. Conclusions

Numerous investigations have revealed the multidirectional role of SIRTs in the major processes underlying CNS disorders. Understanding these mechanisms allows for a more specific diagnostic approach, with the possibility of early recognition of the disease and monitoring its course. Elucidation of SIRTs molecular roles offers a chance to define relevant markers of pathologic processes in CNS and promising candidates for novel therapeutic targets (e.g., activators of SIRT1 and inhibitors of SIRT2). These options seem especially encouraging regarding neurodegenerative disorders, the burden of which is increasing worldwide due to aging societies. Nevertheless, SIRTs, as crucial players in the crosstalk between inflammation, degeneration, and metabolic alterations, seem like a putative target for interventions beneficial in other CNS disorders.

SIRTs-related agents (mainly resveratrol and other activators of SIRT1) have already been tested in clinical trials in the range of diseases, but with ambiguous results. They were shown to have some beneficial effects in diabetes (improved endothelial function and arterial stiffness, modified lipid profile, regulated insulin resistance), cancer (reduced proliferation of tumor cells, suppression of enzymes involved in carcinogenesis), and psoriasis [149]. However, many therapeutic trials failed to provide any significant results. The main challenges associated with the incorporation of findings from experimental studies on SIRTs into clinical ones include complex and bidirectional effects of SIRT activity (“double-edged sword”), genetic and biochemical differences between human diseases and their animal models, and difficulties with identifying candidate molecules with favorable pharmacodynamic profiles and bioavailability [149]. Considering this, further extensive studies and well-designed randomized trials are warranted to verify the diagnostic and therapeutic utility of SIRTs in CNS diseases.

## Data Availability

Not applicable.
